# Supplementary material for: Downstream Link of Vitamin D Pathway with Inflammation Irrespective of Plasma 25OHD3: Hints from Vitamin D-Binding Protein (DBP) and Receptor (VDR) Gene Polymorphisms
Source: Biomedicines. 2025 Feb 6;13(2):385. doi: 10.3390/biomedicines13020385 (PMC11853708; doi:10.3390/biomedicines13020385)
Supplement: Supplementary file 1 [file biomedicines-13-00385-s001.zip › biomedicines-3451782-supplementary.pdf]

Figure S1. (Supplementary)

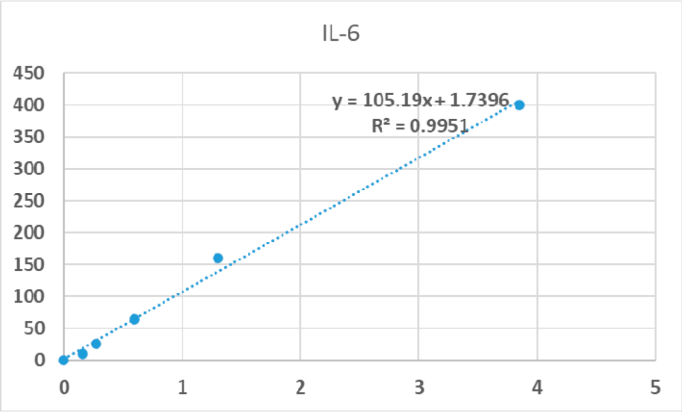

no	Corrected Abs	IL-6 Concentration (pg/mL)
1	0	0
2	0.157941722	10.24
3	0.270382861	25.6
4	0.594823503	64
5	1.302808955	160
6	3.847873713	400

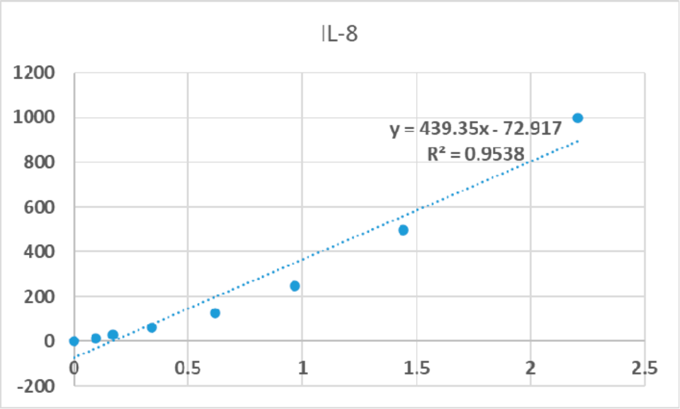

no	Corrected Abs	IL-8 Concentration (pg/mL)
1	0	0
2	0.09641216	15.6
3	0.169772786	31.2
4	0.341330311	62.5
5	0.619810709	125
6	0.968739083	250
7	1.441049262	500
8	2.20709776	1000

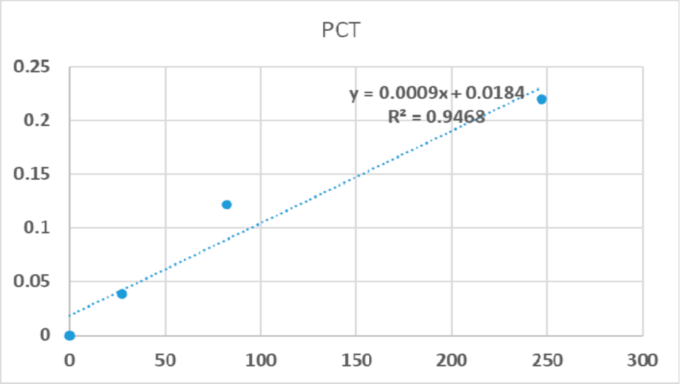

no	Corrected Abs	PCT Concentration (pg/mL)
1	0	0
2	0.03818136	27.43
3	0.12184686	82.3
4	0.22043267	246.9
5	0.59951236	740.7
6	1.37702519	2222
7	1.98383597	6667
8	3.15404516	20000

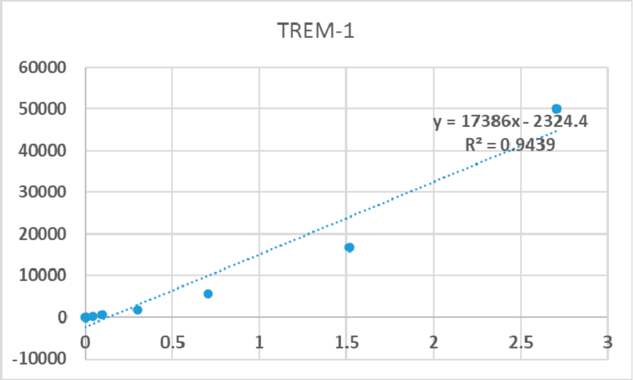

no	Corrected Abs	TREM-1 Concentration (pg/mL)
1	0	0
2	0.00822113	68.59
3	0.046877883	205.8
4	0.097152897	617.3
5	0.302221338	1852
6	0.707929594	5555
7	1.516061875	16667
8	2.703051836	50000

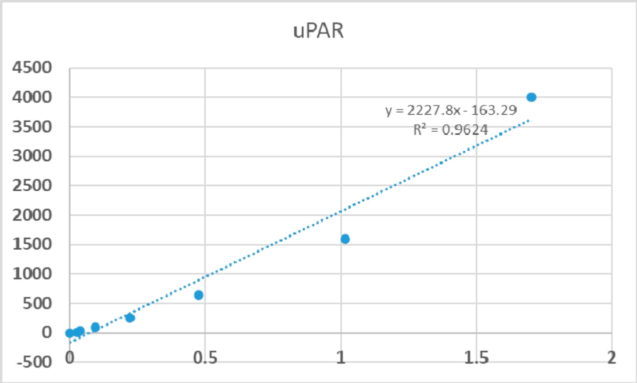

no	Corrected Abs	uPAR Concentration (pg/mL)
1	0	0
2	0.028897158	16.38
3	0.037507344	40.96
4	0.093461362	102.4
5	0.222265168	256
6	0.473911882	640
7	1.014094048	1600
8	1.703783962	4000

Figure S1. Supplementary

The diagrams on the left side and their corresponding table display the standard curve for each tested marker generated by plotting the Absorbance (Abs) of the standards against their known concentrations in pg/mL.
